# Recent infection by *Wolbachia* alters microbial communities in wild *Laodelphax striatellus* populations

**DOI:** 10.1186/s40168-020-00878-x

**Published:** 2020-07-02

**Authors:** Xing-Zhi Duan, Jing-Tao Sun, Lin-Ting Wang, Xiao-Han Shu, Yan Guo, Matsukura Keiichiro, Yu-Xi Zhu, Xiao-Li Bing, Ary A. Hoffmann, Xiao-Yue Hong

**Affiliations:** 1 Department of Entomology, Nanjing Agricultural University, Nanjing, 210095 Jiangsu China; 2 NARO Kyushu Okinawa Agricultural Research Center, 2421 Suya, Koshi, Kumamoto, 861-1192 Japan; 3 School of BioSciences, Bio21 Institute, The University of Melbourne, Melbourne, Victoria 3010 Australia

**Keywords:** *Laodelphax striatellus*, Microbial community, *Wolbachia*, Endosymbiont, Microbial interactions

## Abstract

**Background:**

Host-associated microbial communities play an important role in the fitness of insect hosts. However, the factors shaping microbial communities in wild populations, including genetic background, ecological factors, and interactions among microbial species, remain largely unknown.

**Results:**

Here, we surveyed microbial communities of the small brown planthopper (SBPH, *Laodelphax striatellus*) across 17 geographical populations in China and Japan by using 16S rRNA amplicon sequencing. Using structural equation models (SEM) and Mantel analyses, we show that variation in microbial community structure is likely associated with longitude, annual mean precipitation (Bio12), and mitochondrial DNA variation. However, a *Wolbachia* infection, which is spreading to northern populations of SBPH, seems to have a relatively greater role than abiotic factors in shaping microbial community structure, leading to sharp decreases in bacterial taxon diversity and abundance in host-associated microbial communities. Comparative RNA-Seq analyses between *Wolbachia*-infected and -uninfected strains indicate that the *Wolbachia* do not seem to alter the immune reaction of SBPH, although *Wolbachia* affected expression of metabolism genes.

**Conclusion:**

Together, our results identify potential factors and interactions among different microbial species in the microbial communities of SBPH, which can have effects on insect physiology, ecology, and evolution.

Video Abstract

## Background

The fitness of insects can be affected by their interactions with associated microbiomes [1–3]. Fitness traits affected by host microbiomes include development [4], fecundity [5], resistance to natural enemies [6], climate adaptation [7], and synthesis of essential amino acids [8, 9]. In addition, disturbing an insect’s bacterial population can change host fitness [10], such as producing enhanced sensitivity to bacterial pathogens in bees [11] and altering fecundity in mosquitos [12, 13].

The microbial communities of hosts are influenced by diverse factors that include diet [14], pH [15], host [16], life stage [17], temperature and humidity [18], and genetic background [19]. Evidence for effects of genetic background on microbial communities is mostly based on correlations between microbial structure and phylogenetic relationships at the macro-evolutionary level [20, 21], although such correlations might reflect factors like geographic isolation that drive speciation rather than genetic backgrounds per se. Apart from external factors, changes in microbial communities can also be driven by interactions between different microbial species [22]. For example, *Wolbachia* has been shown to compete against pathogens in *Drosophila* [23] and *Aedes* [24, 25]. Similarly, *Spiroplasma* reduces the density of *Wolbachia* in *Drosophila* [26] and *Asaia* impedes the vertical transmission of *Wolbachia* in *Anopheles stephensi* mosquitoes [27]. Mechanisms involved in these microbial interactions are often not clear.

To understand factors influencing the microbial distribution within hosts, investigations are needed at the population level when there are likely to be fewer confounding effects than in interspecific comparisons across hosts. Here, we undertake such an investigation on the small brown planthopper (SBPH, *Laodelphax striatellus*), a notorious agricultural pest that damages rice plants by sucking rice sap and spreading rice stripe virus (RSV) [28]. The SBPH has a strong migratory ability but also shows population genetic differentiation [29, 30], providing a suitable model for studying the impact of genetic background on microbiomes. Previous studies of the microbiota of SBPH have relied on laboratory samples [31–33]. However, stable laboratory rearing conditions are likely to alter the original microbial community structure which might be shaped by their original environmental conditions, with a homogenizing effect on the microbial community [34–36]. Moreover, genetic drift can occur, affecting the genetic background of both the host and the microbial community during rearing, generating potential differences between the microbial communities observed in the lab and the field. Given these concerns, our current study focusses on natural populations. We combine 16S rRNA amplicon sequencing with a transcriptome analysis to test factors shaping the microbial community in their host at the population level, and we explore the nature of the interactions between different microbial species.

## Methods

### Sample collection

**Fig. 1:**
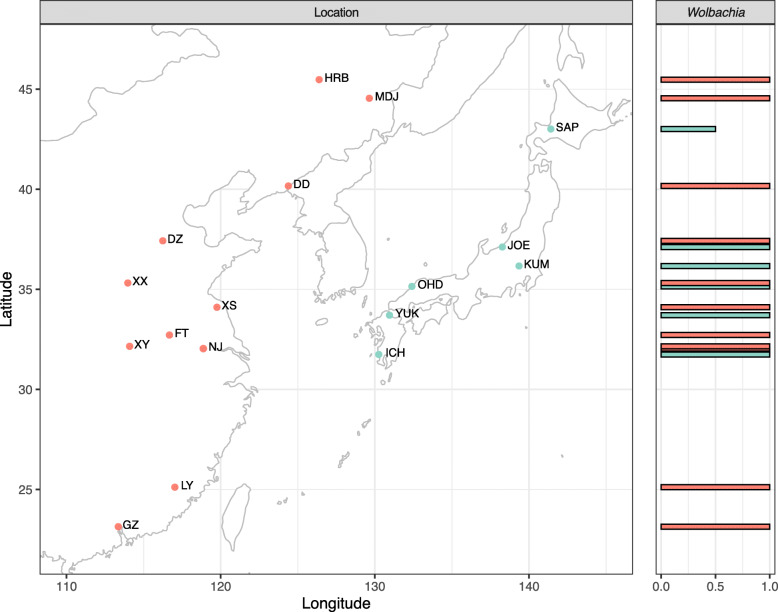
Sampling localities (left) and infection frequencies (right) of *Wolbachia* in natural populations of SBPH across 17 locations. The numbers in the location map indicate the numbers of SBPH detected. Positions of the infection frequency bars correspond to the latitude of the population. The locations and dates of collection are given in Additional file 1: Table S1

SBPH individuals were collected from rice plants at 17 locations in China and Japan during the summers (May to September) of 2010–2018 (Fig. 1, left panel; Additional file 1: Table S1). We haphazardly collected about 60–100 adult female individuals at each location. To avoid sampling siblings, we collected only one SBPH per host plant and selected host plants that were at least 1 m apart. All samples were preserved in 100% ethanol and stored at − 20 °C until DNA extraction.

### 16S rRNA amplicon sequencing

For each of the 17 locations, three female adults were pooled to provide a biological replicate and three biological replicates were established per location. Total genomic DNA was extracted with a DNeasy blood and tissue kit (Qiagen, Hilden, Germany) according to the manufacturer’s protocols. A two-step PCR approach recommended by Illumina [37] was performed to generate amplicon libraries. Briefly, the PCR amplification of the bacterial 16S rRNA genes involved universal primer sets 338F (5′-ACTCCTACGGGAGGCAGCAG-3′) and 806R (5′-GGACTACHVGGGTWTCTAAT-3′). The PCR products were purified on a 2% agarose gel, and extracted with an AxyPrep DNA Gel Extraction Kit (Axygen Biosciences, Union City, CA, USA). The Illumina sequencing adapters and sample-specific barcodes were added to the purified PCR products with a second PCR using the TruePrep Index Kit V3 for Illumina (Vazyme, Nanjing, China). Final PCR products were purified with Hieff NGS DNA selection Beads (YEASEN, Shanghai, China), and equalized and normalized using the dsDNA HS assay kit for Qubit (YEASEN, Shanghai, China). Samples were quantified and pooled in equimolar ratio using a Qubit 4 Fluorometer (Invitrogen, Waltham, MA, USA) and then were submitted to Majorbio Bio-Pharm Technology Company Limited (Shanghai, China) for high-throughput sequencing on an Illumina MiSeq PE300.

After sequencing, raw fastq files were demultiplexed, quality-filtered by Trimmomatic, and merged by FLASH [38] (http://www.cbcb.umd.edu/software/flash). OTUs were clustered with 97% similarity cutoff using UPARSE [39] (version 7.1, http://drive5.com/uparse/) and sequences were then phylogenetically assigned to taxonomic classifications using an RDP classifier [40] (http://rdp.cme.msu.edu/). To normalize sequencing depth, the samples were rarefied to 34135 sequences (the lowest coverage sample) to ensure a random subset of OTUs for all samples.

### Mitochondrial *COI* gene PCR

In SPBH, no significant differentiation among populations exists for nuclear genes but mitochondrial genes that are passed down from mother are differentiated [29]. To determine the degree of genetic differentiation, 20 to 46 female adults were haphazardly selected from each population (Fig. 1, left panel) for mitochondrial *COI* gene amplifications and sequencing according to Sun et al. [29]. The PCR products were sent to Tsingke Biological Technology Company (China) for sequencing.

### Diagnostic PCR

To measure infection frequencies of *Wolbachia*, an additional eight to 46 female adults were haphazardly selected from each population. The specific primers [41] are listed in Additional file 1: Table S2. DNA extraction and PCR were done as described above. Positive controls (known sample with *Wolbachia*) and blank controls were also run. PCR products of 599 bp size were run on 1.0% agarose gels stained with ethidium bromide at 150 volts and visualized by GenoSens 1860 (Clinx, Shanghai, China). The number of samples showing bright DNA bands compared with the DL 2000 DNA mark (Tsingke, China) was used to calculate the infection rate.

### Transcriptome analyses

To investigate the effects of *Wolbachia* infection on the SBPH transcriptome, we compared *Wolbachia*-free and *Wolbachia*-infected SBPH strains. The uninfected strain was obtained by treating the infected strain with tetracycline for 10 generations according to the method of Guo et al. [42]. Briefly, approximately 30 abdomens of SBPH as a biological replicate were dissected from 3-day-adults of both *Wolbachia*-infected and *Wolbachia*-free females. The female abdomens contain a large quantity of fat body and blood cells which are the basis of innate immunity. Total RNA was extracted from three biological replicates using TRIzol Reagent (Invitrogen, CA, USA) according to the manufacturer’s instructions. RNA purity was measured with a NanoPhotometer® spectrophotometer (IMPLEN, CA, USA). RNA concentration was measured with a Qubit® RNA Assay Kit in a Qubit® 2.0 Fluorometer (Life Technologies, CA, USA). Finally, RNA was pooled for Illumina MiSeq sequencing (BGI, Wuhan, China) according to a standard protocol [43].

The sequencing generated 6.6 Gb per biological replicate. Clean reads were obtained by removing reads with adaptors, poly-N, and having a low quality. Gene expression levels were estimated by RSEM software package [44] (http://deweylab.biostat.wisc.edu/rsem). Immune-related genes of SBPH were obtained from Zhu et al. [45], which were generated by alignments with immune genes of *D*. *melanogaster*, *A*. *gambiae*, *Aedes aegypti*, and *Culex quinquefasciatus* by using BLASTX [46]. In addition, sequences were annotated to the KO database with the KEGG Automatic Annotation Server.

### Statistical analyses

Bray–Curtis dissimilarity metrics among all samples were constructed using *beta_diversity*.*py* in QIIME [47] (http://qiime.sourceforge.net/) and were visualized with a principal coordinate analysis (PCoA). The difference of microbial communities among the populations was calculated by ADONIS. The population genetic differentiation value (*F*_ST_) was calculated in Arlequin 3.5 [48]. The annual mean temperatures (Bio1) and the annual mean precipitation (Bio12) of the 17 locations were obtained from DIVA-GIS 7.5.0 [49] (https://www.diva-gis.org), which is a geographic information system for the analysis of species distribution data. A structural equation model (SEM) [50] was used to estimate the relative contributions of *F*_ST_, Bio1, Bio12, latitude, and longitude (Additional file 1: Table S3; Table S4; Table S1) on microbial community structure with communities based on Bray–Curtis dissimilarity metrics. The SEM tests were performed in the R “SEM” package (https://cran.r-project.org/web/packages/sem/index.html), and the path diagram for the SEM tests is shown in Fig. 4. As non-normal distribution of variables may compromise SEM analyses results, we also undertook Mantel tests using the Spearman method with 1000 permutations to determine the associations between microbial community structure variation and the five aforementioned factors.

The relative abundance of a given phylogenetic group was estimated by examining the number of reads of that group for each population. In order to analyze the evenness and richness of the microbial community, we calculated several α diversity indexes including the Sods, Shannon, Simpson, Ace, Chao, and Coverage indexes. Spearman’s rank correlations were calculated between the proportion of *Wolbachia* and the α diversity indexes (Shannon indexes and Simpson indexes) of the populations. The significance of differences in read proportions of bacterial 16S rRNA genes at the genus level was assessed by Mann–Whitney *U* tests. The significance of differences in α diversity indexes between *Wolbachia*-infected and -uninfected populations was calculated by a *t* test. All statistical analyses were carried out in R 3.5.2 [51].

### Probabilistic features recognition for the OTU distribution

**Fig. 2:**
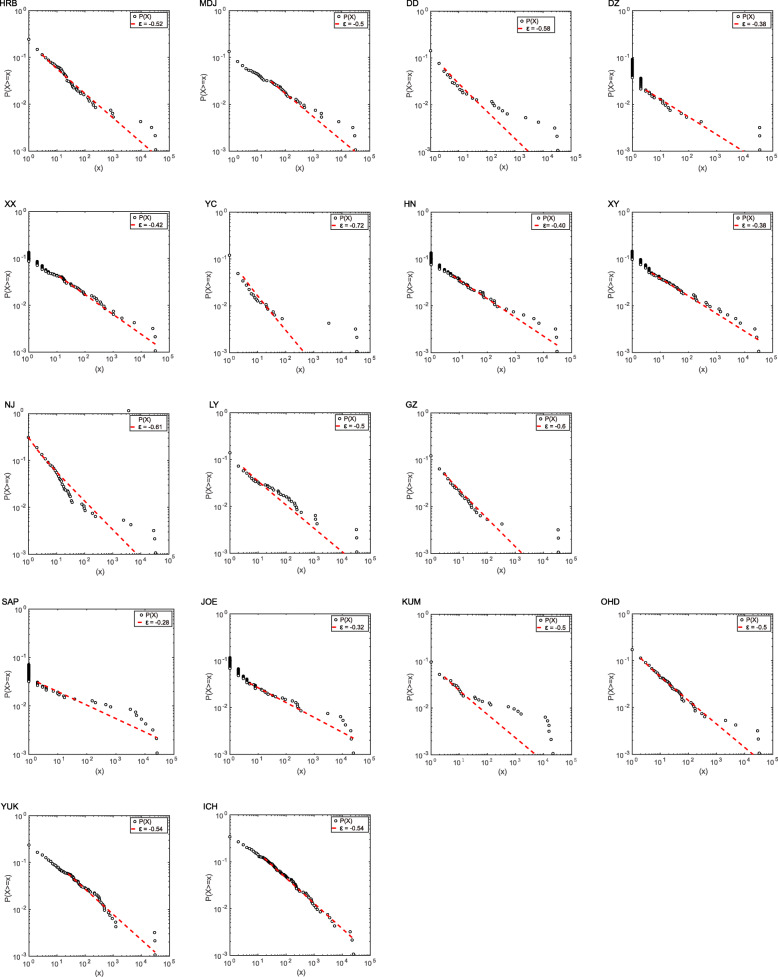
Exceedance probability distribution function of OTU abundance for each population. A power-law function is used as the model to estimate the pdf of abundances. Population codes are given in Additional file 1: Table S1

Components of collective ecological and biological systems presented an obvious probabilistic similarity in their aggregation, in which only several species made up a relatively high share of the whole sample, while most species accounted for much less. By looking into our datasets, we noted that the abundance data of OTUs explicitly met this property. Therefore, the power-law function that satisfied the mathematical characterization of such distribution behavior was considered as an appropriate function to recognize the probability distribution features of OTUs. Given the type of power-law function, the abundance had the probability density function (pdf):

1.1 $$ p(x)={ax}^{-\varepsilon },x>x' $$

where *x*’ was the threshold that ensured a robust fitting for the power-law distribution. We probabilistically characterized the distribution of abundance of OTUs by calculating the exceedance probability distribution function [52] that was given by:

1.2 $$ P\left(X\ge x\right)={x}^{1-\varepsilon }f\cdot \left(\frac{x}{x\hbox{'}}\right) $$

where *ε* was the scale exponent of power-law distribution underlying the statistical patterns of data considered. This scale factor implied the property of mean and variance of data: when *ε ≤* 2, the mean and variance were both infinite; when 2 < *ε* < 3, the mean existed, while the variance was still infinite; and when *ε ≥* 3, both mean and variance existed. Additionally, $f\left(\frac{x}{x\hbox{'}}\right)$ was introduced to give a general formulation for the homogeneity function. The probabilistic features for the OTU distribution for each population were given in Fig. 2.

To assess the microbial community variations between populations in terms of probabilistic distributions of OTUs, we calculated the Kullback–Leibler divergence (KL divergence) by using the R package “LaplacesDemon” (https://cran.rproject.org/web/packages/LaplacesDem on/index.html). Probability density functions of OTUs used as the arguments for KL divergence calculation function were computed by using the R package “histogram” (https://cran.r-project.org/web/packages/histogram/index.html). The KL divergence was used as a surrogate index of microbial community structure and was also used for SEM and Mantel tests to analyze the relationships between microbial community structure and five putative predictor variables as mentioned above.

## Results

### Microbial diversity and environmental factors in the absence of *Wolbachia*

Based on the infection frequencies of *Wolbachia*, only the SAP population was found to have *Wolbachia*-uninfected individuals. And a notable difference in microbial community structure was found between SAP and the remaining populations as showed by the probabilistic features of the OTU distribution (Fig. 2). To eliminate the potential influence of *Wolbachia* on pooled samples, the SAP population was excluded for testing the impact of other factors on the microbial community. Among the 48 samples from the remaining 16 SBPH populations, the RDP classifier identified a total of 314 OTUs (Additional file 2: Table S5). *Wolbachia* were the most abundant bacteria, accounting for 87.9% of the 16S rRNA gene reads in the Chinese populations and 66.4% of the 16S rRNA gene reads in the Japanese populations (Additional file 1: Table S6). Other prominent genera included *Spiroplasma* (3.55%), *Asaia* (2.47%), *Pantoea* (1.04%), and *Herbaspirillum* (1.03%) in the Chinese populations and *Diplorickettsia* (10.9%), *Asaia* (5.56%), *Spiroplasma* (5.00%) and *Pantoea* (2.08%) in the Japanese populations. Genera other than *Wolbachia* were enriched in the Japanese populations compared with the Chinese populations. These results suggest that the structures of SBPH-associated bacteria were different between the two countries.

**Fig. 3:**
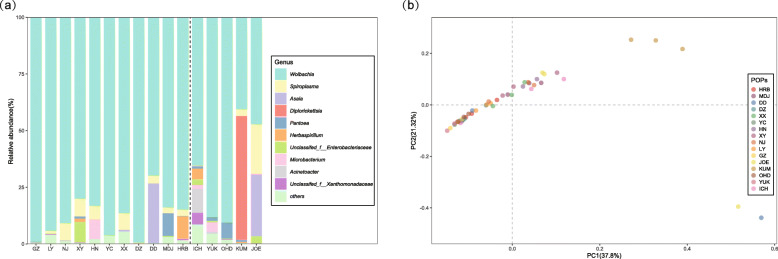
The abundance and distance of microbial communities of SBPH across 16 populations. **a** Relative abundance of bacterial 16S rRNA genes at the genus level. Dashed line separates the Chinese and Japanese microbial community abundance. Blocks of populations were arranged by origin sites (south to north). Other genera (“others”) account for < 5% of the classified sequences. **b** Principal coordinate analysis (PCoA) of SBPH samples collected from different locations. PCoA was generated by the Bray–Curtis dissimilarity method

**Fig. 4:**
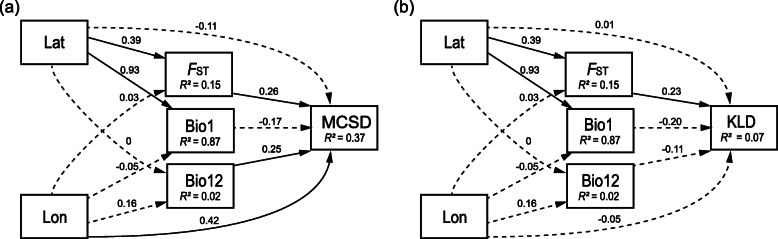
Path diagram for the structural equation model (SEM) for **a** environmental/genetic factors and microbial Bray–Curtis dissimilarity, and **b** environmental/genetic factors and KL divergence in natural populations of SBPH. Statistically significant positive paths are indicated by solid arrows. Statistically significant negative paths are indicated by dashed arrows. The *R*^2^ values in each box indicate the amount of variation in that variable explained by the input arrows. Numbers next to arrows are unstandardized slopes. Lat, Latitude; Lon, Longitude; MCSD, microbial Bray–Curtis dissimilarity; KLD, KL divergence

We found significant differences in microbial communities among the 16 populations (Fig. 3b) based on Bray–Curtis dissimilarity (ADONIS, *r* = 0.428, *p* = 0.001) and considerable variations based on probabilistic features of the OTU distribution (Fig. 2). To understand whether and to what extent host genetic and environmental factors contributed to variation in microbial communities across the populations, structural equation model (SEM) was used to resolve the relationships between microbial community structure and five putative predictor variables *F*_ST_, Bio1, Bio12, latitude, and longitude. The results showed that differences in the microbial community structure characterized by Bray–Curtis dissimilarity could be significantly explained by longitude and annual mean precipitation (Bio12), suggesting geographical location and precipitation help shape the microbial community structure in SBPH (Fig. 4a; Additional file 1: Table S7). However, no significant association between KL divergence based on the probability densities of OTUs and any geographic or environmental factors was detected by SEM and the Mantel tests (Fig 4b; Table 1; Additional file 1: Table S7).

**Table 1:** Effects of factors in the Mantel test analysis undertaken on 16 populations where *Wolbachia* was fixed in the population

Variation	Effect	*r*	*P*
Microbial Bray–Curtis dissimilarity	Genetic differentiation (*F*_st_)	0.162	0.153
	Latitude	− 0.106	0.678
	Longitude	0.409	0.001
	Annual mean temperature (Bio1)	− 0.051	0.564
	Annual precipitation (Bio12)	0.306	0.029
KL divergence	Genetic differentiation (*F*_st_)	0.163	0.089
	Latitude	0.006	0.419
	Longitude	− 0.057	0.683
	Annual mean temperature (Bio1)	0.034	0.311
	Annual precipitation (Bio12)	− 0.095	0.848

Pairwise *F*_ST_ values computed from the mitochondrial *COI* gene (887 bp) for the 16 populations showed that 64 of the 120 pairwise population comparisons were significantly different (Additional file 1: Table S3). The SEM analyses also showed significant effects of *F*_ST_ on the microbial Bray–Curtis dissimilarity (Fig. 4a; Additional file 1: Table S7), suggesting that mtDNA background correlated with similarity in the microbial community structure among populations. In addition, latitude was found to be associated with *F*_ST._ In line with the SEM analyses, Mantel tests showed that longitude and Bio12 significantly correlated with microbial Bray–Curtis dissimilarity (Table 1). However, although an effect of *F*_ST_ was detected in the SEM, the correlation from the Mantel test was not significant (*r* = 0.162, *p* = 0.153). This may reflect the lower sensitivity of the Spearman method and reduced effect of genetic background relative to the other two factors. For the analyses based on KL divergence, the SEM analyses showed that KL divergence significantly correlated with *F*_ST_ values (Fig. 4b; Additional file 1: Table S7) but the Mantel test was marginally non-significant (Table 1).

### Effects of *Wolbachia* on population variation in microbial communities

The 16S rRNA gene data revealed microbial community structure across populations at the genus level (Fig. 3a). The proportions of *Wolbachia* reads in the high latitude populations of Japan (KUM, JOE, and SAP) were low. To test whether the spread of *Wolbachia* might affect microbial community structure, diagnostic PCR was conducted to assess the frequency of *Wolbachia* across the 17 populations (Fig. 1, right panel). The results showed that the infection rate of *Wolbachia* in SAP was 50% while it was 100% in the other populations. While these results are similar to previous findings in SBPH showing a relatively higher *Wolbachia* incidence in China [53], the frequency of *Wolbachia* infection observed in the present study was higher than it was in the previous studies, especially in Japan [54]. This showed that *Wolbachia* has increased in recent decades.

**Fig. 5:**
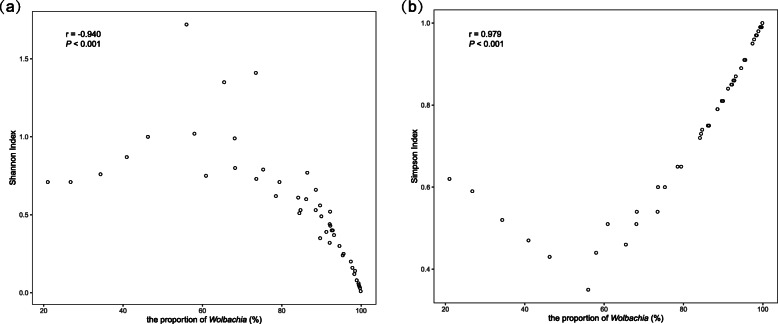
Relationships between the proportions of *Wolbachia* and the Shannon indexes (**a**) or the Simpson indexes (**b**) of microbial community among the 51 samples by Spearman’s rank correlation. *R* values and *P* values of each linear regression plots are provided

The correlations between the α diversity indexes (Shannon and Simpson indexes) [55, 56] and the proportion of *Wolbachia* in all samples were examined by Spearman’s rank correlation analyses (Fig. 5a, b). The results revealed that the proportions of *Wolbachia* were significantly correlated with the Shannon (*r* = − 0.940, *P* < 0.001) and Simpson (*r* = 0.979, *P* < 0.001) indexes, suggesting that the presence of *Wolbachia* in SPBH decreased the richness and evenness of microbial communities.

### *Wolbachia* infection and the relative abundance of bacterial taxa in SPBH

**Fig. 6:**
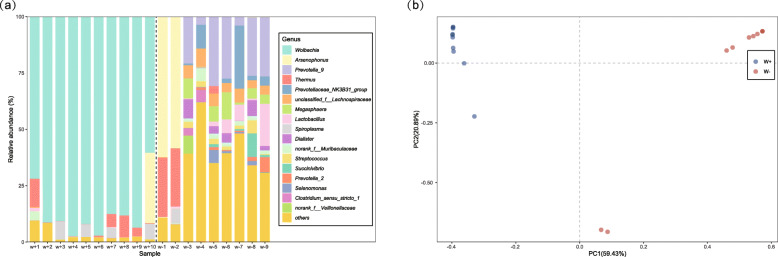
The abundance and distance of microbial community of SBPH between *Wolbachia*-infected and *Wolbachia*-free female adults. **a** Relative abundance of bacterial 16S rRNA genes at the genus level from 10 *Wolbachia*-infected female adults and 9 *Wolbachia*-free female adults in SAP population. Dashed line separates the microbial community abundance of the two groups. Other genera (“others”) account for < 5% of the classified sequences. **b** Principal coordinate analysis (PCoA) among *Wolbachia*-infected and *Wolbachia*-free female adults. PCoA was generated by the Bray–Curtis dissimilarity method

**Fig. 7:**
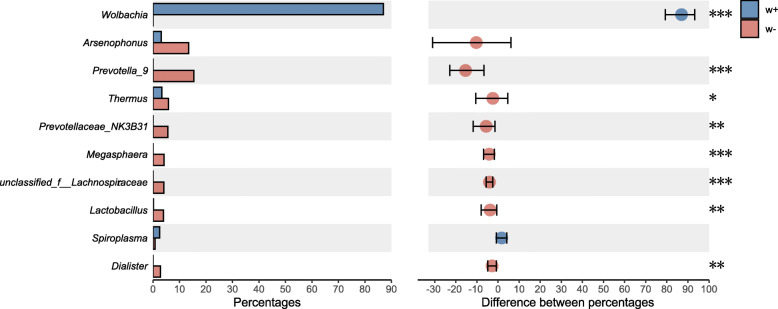
Read percentages of bacterial 16S rRNA genes among *Wolbachia*-infected and *Wolbachia*-free female adults at the genus level. Data were showed as relative abundance (%) of genus. Statistical analysis was performed by the Mann–Whitney *U* test. Error bars represent 95% confidence intervals. *P < 0.05, **P < 0.01, ***P < 0.001 and w+ vs. w−

To further test the impact of *Wolbachia* infection on the microbial communities, 10 female adults infected with *Wolbachia* and 9 female adults uninfected with *Wolbachia*, both from the SAP population, were used to compare the microbial communities by 16S rRNA amplicon sequencing. After the samples were rarefied to 39,872 sequences (the lowest coverage sample), 1985 OTUs were obtained between the two groups (Additional file 2: Table S8). *Wolbachia* predominated in the microbial communities of *Wolbachia*-infected females (Fig. 6a). The relative abundances of 154 genera in the *Wolbachia*-infected adults were significantly reduced (Additional file 2: Table S9). PCoA analysis based on Bray–Curtis dissimilarity (Fig. 6b) clearly separated the *Wolbachia*-infected individuals from the *Wolbachia*-uninfected individuals, indicating that the microbial community structures of the two groups were significantly different. Compared to the *Wolbachia*-infected group, the *Wolbachia*-free group possessed high microbial diversity as suggested by the Sobs, Shannon, Simpson, Chao. and Ace indexes (Table 2: Welch two-sample *t* test: *p* = 0.003 for Sobs, *p* < 0.001 for Shannon, *p* < 0.001 for Simpson, *p* = 0.001 for Chao, *p* = 0.002 for Ace). Furthermore, Mann–Whitney *U* tests revealed that the abundances of seven genera that dominated the communities found in the *Wolbachia*-free adults were very low in the *Wolbachia*-infected adults (Fig. 7). These results provided further evidence that *Wolbachia* decreased the relative abundance and diversity in the microbial community of SBPH.

**Table 2:** Measures of species richness and evenness of SBPH from 10 *Wolbachia*-infected females and 9 *Wolbachia*-free females from the SAP population

Samples	Sobs	Shannon	Simpson	Ace	Chao	Coverage
w+1	693	1.594	0.532	816.372	766.533	0.996
w+2	356	0.707	0.832	434.097	409.182	0.998
w+3	76	0.378	0.829	206.037	126.647	0.999
w+4	161	0.210	0.949	234.636	206.217	0.998
w+5	139	0.406	0.850	188.332	178.200	0.999
w+6	132	0.206	0.943	314.430	235.542	0.998
w+7	107	0.561	0.771	199.706	152.000	0.999
w+8	95	0.475	0.787	209.990	159.688	0.999
w+9	96	0.337	0.877	148.918	133.625	0.999
w+10	93	0.965	0.466	149.309	143.167	0.999
w−1	247	1.232	0.459	342.134	312.632	0.998
w−2	402	1.490	0.412	431.463	443.143	0.998
w−3	399	3.987	0.040	461.191	457.400	0.998
w−4	636	5.063	0.013	689.927	684.838	0.998
w−5	528	3.990	0.052	628.688	643.000	0.997
w−6	516	3.962	0.052	630.807	621.726	0.997
w−7	640	4.590	0.027	709.431	722.787	0.997
w−8	534	3.915	0.052	632.849	651.018	0.997
w−9	497	3.817	0.065	599.893	629.255	0.997

### Changes in microbial communities by *Wolbachia* infection

**Fig. 8:**
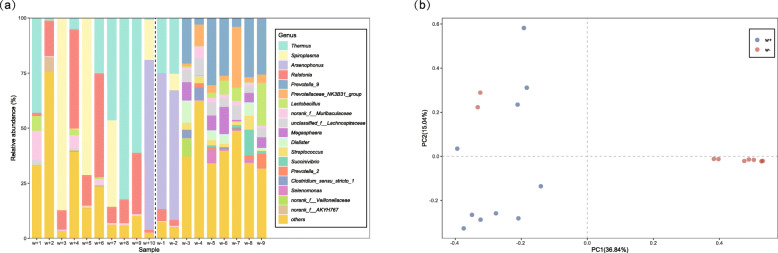
Abundance and distance of microbial community of SBPH between *Wolbachia*-infected (excluding *Wolbachia* reads) and *Wolbachia*-free female adults. **a** Relative abundance of bacterial 16S rRNA genes at the genus level from 10 *Wolbachia*-infected female (excluding *Wolbachia* reads) adults and 9 *Wolbachia*-free female adults in SAP population. The dashed line separates the microbial community abundance of the two groups. Other genera (“others”) account for < 5% of the classified sequences. **b** Principal coordinate analysis (PCoA) among *Wolbachia*-infected (excluding *Wolbachia* reads) and *Wolbachia*-free female adults. PCoA was generated by the Bray–Curtis dissimilarity method

**Fig. 9:**
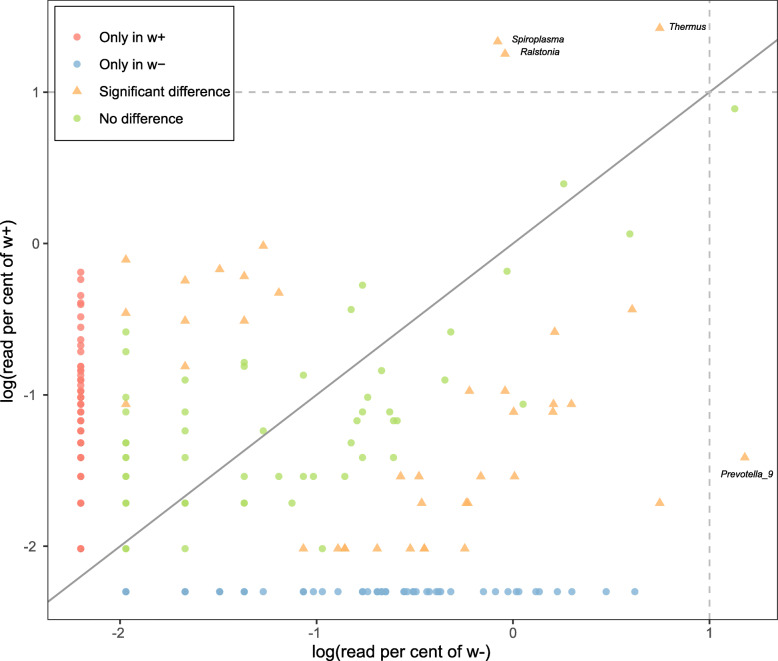
Common logarithm values of the read percentage of bacterial 16S rRNA genes for each genus across the microbial communities between *Wolbachia*-infected females (excluding *Wolbachia* reads) and *Wolbachia*-uninfected females and comparisons by Mann–Whitney *U* tests. Significant differences in the genera existing in *Wolbachia*-infected females (excluding *Wolbachia* reads) and *Wolbachia*-uninfected females are indicated by different colors. Proportions where genera in *Wolbachia*-infected females (excluding *Wolbachia* reads)/*Wolbachia*-uninfected females = 1 is shown as a dotted line

To detect the effect of *Wolbachia* infection on the structure of the microbial community, we compared microbial taxon abundance between the *Wolbachia*-infected and *Wolbachia*-free individuals sampled from the SAP population. To normalize sequencing depth, we haphazardly extracted 1144 reads for each sample (based on the minimum number of reads after removing *Wolbachia* reads in the *Wolbachia*-infected samples, Additional file 1: Table S10) for these analyses. Our results showed that the structures of the microbial communities were different between *Wolbachia*-infected females (after excluding *Wolbachia* reads) and *Wolbachia*-uninfected females (Fig. 8a; Additional file 1: Table S11). Both the Shannon and Simpson indexes indicated that the *Wolbachia*-free group possessed higher microbial diversity than *Wolbachia*-infected group (excluding *Wolbachia* reads) (Additional file 1: Table S12; Welch two-sample *t* test: *p* < 0.035 for Shannon, *p* = 0.020 for Simpson). PCoA analysis based on Bray–Curtis dissimilarity (Fig. 8b) also clearly separated the two groups, except for two samples of the *Wolbachia*-uninfected females. Two samples contained very few *Wolbachia* reads (accounting for 0.04% of their microbial communities), which might lead to a distorted pattern. However, it appears that even *Wolbachia* infections at low titers can significantly change the microbial community. In addition to decreasing bacterial diversity, we also found that the *Wolbachia* infection changed bacterial taxon abundance, with 25 genera significantly increasing and 65 significantly decreasing in *Wolbachia*-infected individuals (Fig. 9; Additional file 2: Table S13; Table S14; Table S15). Most of these bacteria have widespread distributions in insect tissues, including the gut, ovary, and head. Notably, four genera occurring in high proportions (with log (read percent) > 1) in both *Wolbachia*-infected and *Wolbachia*-free groups were also significantly different, with *Thermus*, *Spiroplasma*, and *Ralstonia* enriched in the *Wolbachia*-infected group, in contrast to *Prevotella_9* which was enriched in the *Wolbachia*-uninfected group (Fig. 9). Apart from these changes, *Wolbachia* infection seems associated with the existence of particular bacterial taxa, with 160 genera specifically existing in relative low abundance in the *Wolbachia*-infected group (Additional file 2: Table S13).

### *Wolbachia* does not appear to strongly affect immune-related genes of SBPH but affects metabolism genes

**Fig. 10:**
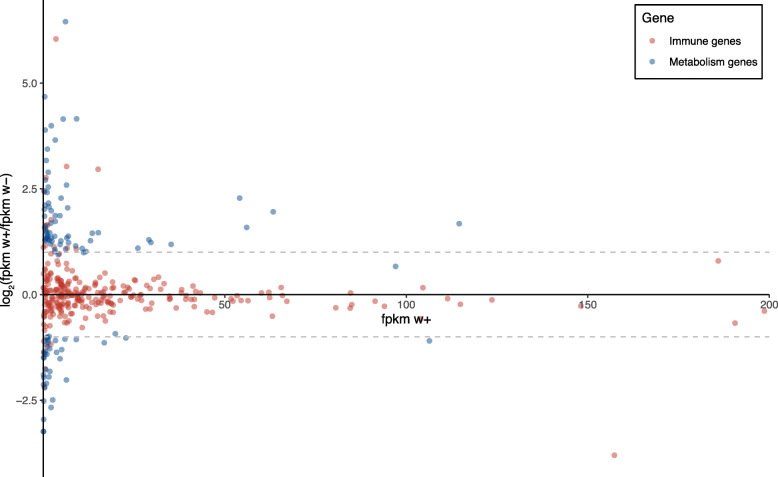
Effects of *Wolbachia* on immune and metabolism genes. Differential expression analysis of immune-related genes and metabolism genes between *Wolbachia*-infected and *Wolbachia*-free female adults expressed in the abdomens. Immune-related genes of SBPH were obtained from Zhu et al. [45] and metabolism genes were obtained by KEGG. The *x*-coordinate shows fpkm value of the *Wolbachia*-infected females, and the *y*-coordinate shows the log_2_(fpkm w+/fpkm w−) value. Dotted lines show the 1 and − 1 values of the *y*-coordinate. To make the results more intuitive, points were excluded where w+ FPKM was greater than 200, including 19 non-significantly expressed immune genes (Additional file 2: Table S16) and one significantly expressed metabolic gene (Additional file 2: Table S17)

To test if *Wolbachia* promotes the expression of immune-related genes in SBPH, we compared the transcriptomes of pooled abdomens from *Wolbachia*-infected and *Wolbachia*-free females. Of 330 immune-related genes in SBPH identified by Zhu et al. [45], 306 genes representing 25 gene families were identified (Additional file 2: Table S16). Most of these genes were not differentially expressed (Fig. 10; Additional file 2: Table S16), which suggests that *Wolbachia* had little or no impact on the immune systems of SBPH. However, through an analysis of Kyoto Encyclopedia of Genes and Genomes (KEGG) terms, we found 141 differentially expressed genes in metabolism processes including oxidative phosphorylation-related and glycolysis-related genes (Fig. 10; Additional file 2: Table S17), which suggests that the effect of *Wolbachia* on microbial community is likely mediated through changing the overall metabolism and physiology of SBPH.

## Discussion

### Effects of environmental factors and genetic background on the microbial community of SBPH

Our analyses suggest that, based on Bray–Curtis dissimilarity, longitude and precipitation may impact microbial communities, and these effects appear separate because precipitation did not associate with longitude. To date, any effects of precipitation on insect microbiome have rarely been considered. Our previous study in spider mites found that precipitation can influence the incidence of *Spiroplasma* [57], a facultative endosymbiont which can manipulate host production. As the SBPH is polyphagous, any effects of longitude and precipitation may reflect effects of these variables on vegetation and food resources for SBPH, which could alter the physiology and metabolism of SBPH hosts and in turn influence microbial communities. SBPH might acquire some bacteria directly from plant sap, as has been suggested for the cochineal insect *Dactylopius* [58], and different bacteria present in different environments could contribute to variation in microbial communities. For example, *Pantoea* was abundant in the MDJ population of SBPH (Fig. 3a) and is thought to have been acquired from the environment in *Ae*. *albopictus* [59]. It is also possible that microbial communities are responding directly to environmental factors rather than being acquired from the environment, and they might even provide a fitness advantage to hosts under certain conditions, although this remains speculative in the absence of experimental data. Future studies should also consider the impacts of variability in climatic variables on microbial communities, whereas we have only considered the average estimates available to us from the tested locations.

Our results based on both Bray–Curtis dissimilarity and KL divergence suggested an association between mtDNA variations and microbial community structure. Previous studies at the macro-evolutionary level have suggested associations between mtDNA variation and microbial communities, but these might reflect geographic isolation that drive speciation rather than genetic backgrounds per se [20, 21], whereas our findings from the population level with shallow divergence in the mitochondrial genome [30] provide relatively more direct evidence of an association. Some bacterial groups that are maternally transmitted and living inside cells (like *Wolbachia*) might be expected to be associated with mtDNA variants which can hitchhike along with spreading endosymbionts [60]. A more recent study in mice found that different mitochondrial genotypes can alter ROS productions, which modulates microbial structure in the host gut [61]. In SBPH, two mitochondrial haplogroups thought to be associated with altered functions exist in natural populations [30], and their impacts on microbial communities could be explored in future work.

### The effects of *Wolbachia* on the microbial community of SBPH

Maternally inherited *Wolbachia* endosymbionts are common in insects. They can manipulate host reproduction, facilitating *Wolbachia*’s rapid spread in a host population. In SBPH, *Wolbachia* can induce strong cytoplasmic incompatibility (CI), resulting in no offspring when uninfected females mate with infected males [62]. Comparison of the microbial communities of *Wolbachia*-free and *Wolbachia*-infected SBPH individuals clearly shows that *Wolbachia* infection severely decreases the diversity and abundance of bacteria in SBPH. The abundance of the seven other main genera in *Wolbachia*-infected adults was very low (Fig. 7). A similar phenomenon has been observed in *Aedes aegypti*, in which a large proportion of bacterial taxa disappeared when *Wolbachia* was induced by artificial injection [63]. Bacterial diversity was also found to be very low in the gut of *Drosophila melanogaster*, which is naturally infected with *Wolbachia* [64].

Significant differences in microbial communities were observed between the Chinese and Japanese populations of SBPH (Fig. 3b). The present results, together with previous studies, suggest that *Wolbachia* has rapidly spread in SBPH populations during recent decades in both China and Japan. The incidence of *Wolbachia* has increased from around 90% in Chinese populations [53] to 100% [29], and from around 65% in Japanese populations [54] to more than 90%. The strong CI of *Wolbachia* and the high migratory ability of SBPH likely contribute to this rapid spread. The spread of *Wolbachia* seems to have pushed the infection to fixation in the Chinese populations, while the invasion is still ongoing in the Japanese populations. In Japan, spread is most noticeable in high latitude regions where *Wolbachia* was previously rare. The difference in histories of *Wolbachia* between China and Japan may be contributing to divergence in their SBPH microbial communities, but this remains to be tested directly, such as through comparisons of the communities when hosts are reared in a common environment.

By removing *Wolbachia* reads from the *Wolbachia*-infected females in SAP populations, we further analyzed the effect of *Wolbachia* on the other bacteria and found that *Wolbachia* infection changed microbial evenness and other measures of microbial diversity. Three bacteria (*Thermus*, *Spiroplasma*, *Ralstonia*, Fig. 9; Additional file 2: Table S14) were highly enriched in the *Wolbachia*-infected samples. Vitamin B can be synthesized by *Thermus* [65], as well as by *Wolbachia* where it can lead to an increase in host fertility [66]. *Thermus* associated with *Wolbachia* may provide an intermediate for the synthesis of vitamin B. In *Drosophila neotestacea*, *Wolbachia* can promote the abundance of *Spiroplasma* [67], pointing to the possibility of direct interactions among microbes. On the other hand, the effect of *Wolbachia* on *Spiroplasma* may lead to different tissue tropisms [26] and asymmetrical interactions between the two bacteria where *Spiroplasma* negatively affects the population of *Wolbachia*, but *Wolbachia* does not influence the population of *Spiroplasma* [26]. In SBPH, *Spiroplasma* was found to induce late male killing [68] which is predicted to have advantages not only in facilitating maternal transmission, but also in promoting horizontal transmission of *Spiroplasma*. This is based on the notion that dead males could burst and release *Spiroplasma* spores into the environment [69]. Whether the bursting of dead males also promotes the spread of other microbes such as *Wolbachia* in nature is unclear. If so, it could partly contribute to the rapid spread of CI inducing *Wolbachia* in SBPH populations without decreasing mitochondrial DNA diversity [29]. *Ralstonia* is a devastating soil-borne plant pathogen and affects growth and development of 200 host species belonging to more than 50 botanical families [70]. For SBPH, we speculated that *Ralstonia* may have been obtained from food resources, but the function of *Ralstonia* in insect hosts is unknown. We also note that many bacteria were reduced by *Wolbachia* infections (Additional file 2: Table S15), and most of them were located in the gut, ovary, and head where *Wolbachia* exist [33]. *Wolbachia* may interact competitively with many components of the microbial community of SPBH but this remains to be investigated.

The main mechanisms by which *Wolbachia* are thought to decrease the microbial diversity are immune system modulation and resource competition [63]. Other mechanisms may include *Wolbachia*-induced changes in ROS, transcription/posttranscription, and pH [64]. Because no significant difference in the expressions of immunity-related genes was detected in the transcriptomes of *Wolbachia*-infected and *Wolbachia*-free female adults, it appears that immune modulation is not involved in SBPH. The only effect detected in this study was a decrease in the expression of the gene encoding defensin in the *Wolbachia*-infected females (Additional file 2: Table S16), the opposite of what might be expected. Through KEGG analysis, we showed 141 differentially expression genes involving metabolic processes including oxidative phosphorylation and glycolysis (Additional file 2: Table S17), which may suggest that intracellular localized somatic *Wolbachia* affect the overall metabolism and physiology of the insect to suppress the diversity/abundance of bacterial populations.

*Wolbachia* infection-associated immune regulation has been reported in organisms in which *Wolbachia* was artificially introduced [24, 25, 71], but not in organisms that are naturally infected with *Wolbachia* [23, 72]. It seems that immune regulation mediated by *Wolbachia* is lost with a long history of *Wolbachia* colonization. The initial colonization of *Wolbachia* may trigger an immune response in the host, which then changes after long-term co-evolution between *Wolbachia* and its host. If that is the case, managing insect pests by releasing insects artificially infected with *Wolbachia* should be undertaken with caution because the “pathogen blocking” efficiency of insect vectors may eventually be lost in nature as *Wolbachia* and its host co-evolve. We agree with Simhadri et al. [64] who argue that future studies of *Wolbachia*-associated phenotypes should consider the effects of *Wolbachia* on the microbial community.

## Conclusions

In this study, by profiling 16S rRNA genes using next-generation sequencing, we explored the relative contributions of genetic background, ecological factors, and interactions among microbial species on the microbial communities of natural populations of SBPH. Our results suggest that *Wolbachia* infection has a stronger role in shaping the microbial community than ecological factors and genetic (mtDNA) background. When *Wolbachia* is introduced into the community, it seems to become the dominant species and decreases microbial diversity. Comparative RNA-Seq analyses between *Wolbachia*-infected and -uninfected strains indicate that the *Wolbachia* do not seem to alter the immune reaction of SBPH, although *Wolbachia* affected expression of metabolism genes, suggesting *Wolbachia* affect the overall metabolism and physiology of the insect to suppress the diversity/abundance of bacterial populations.

## Supplementary information

**Additional file 1: Table S1** Summary of collection details. The population code (ID), province, city, county, latitude, longitude, and date of the field collections assessed here are provided. **Table S2** Specific primers used in PCR for this study. **Table S3** Pairwise *F*_ST_ estimates between populations based on a sequence of the mitochondrial *COI* gene. Population codes are given in Table S1. **Table S4** Annual mean temperatures (Bio1) and the annual mean precipitation (Bio12) of the 17 locations obtained from DIVA-GIS 7.5.0. **Table S6** Relative abundance of bacterial 16S rRNA genes at the genus level observed for Chinese, Japanese and all populations. **Table S7** Effects of factors in the structural equation model (SEM) analysis undertaken on 16 populations where *Wolbachia* was fixed in the population. **Table S10** After *Wolbachia* was excluded from the *Wolbachia*-infected adults, the composition of all samples from SAP populations. **Table S11** Relative abundance of bacterial 16S rRNA genes at the genus level observed for *Wolbachia*-infected females (after removal of *Wolbachia* reads), *Wolbachia*-uninfected females and all samples. **Table S12** Measures of species richness and evenness of SBPH from 10 *Wolbachia*-infected females (excluding *Wolbachia* reads) and 9 *Wolbachia*-free females from the SAP population.

**Additional file 2: Table S5** Abundance of OTUs among the 48 samples. **Table S8** Abundance of OTUs between the *Wolbachia*-infected and *Wolbachia*-free females in SAP populations. **Table S9** Read proportions of bacterial 16S rRNA genes among *Wolbachia*-infected females and *Wolbachia*-free females at the genus level by Mann-Whitney U tests. **Table S13** Read proportions of bacterial 16S rRNA genes for *Wolbachia*-infected females (excluding *Wolbachia* reads) at the genus level by Mann-Whitney U tests. **Table S14** Significantly increased read proportions of bacterial 16S rRNA genes and the tissue in which their corresponding bacteria were distributed for *Wolbachia*-infected females (excluding *Wolbachia* reads) at the genus level by Mann-Whitney U tests. The tissue distribution of bacteria was obtained from Zhang *et al*. [33]. **Table S15** Significantly reduced read proportions of bacterial 16S rRNA genes and the tissue in which their corresponding bacteria were distributed for *Wolbachia*-infected females (excluding *Wolbachia* reads) at the genus level by Mann-Whitney U tests. The tissue distribution of bacteria was obtained from Zhang *et al*. [33]. **Table S16** Expression differences of immune genes between *Wolbachia*-infected and *Wolbachia*-cured female adults expressed in abdomens. **Table S17** Expression differences of metabolism genes between *Wolbachia*-infected and *Wolbachia*-cured female adults expressed in abdomens.

## Data Availability

The raw reads of 16S rRNA sequencing have been deposited in the NCBI Sequence Read Archive (SRA) database (accession number SRP238740). The raw reads of transcriptomes have been deposited in the NCBI SRA database (accession number SRP195568).
