# Supplementary material for: Recent infection by Wolbachia alters microbial communities in wild Laodelphax striatellus populations
Source: Microbiome. 2020 Jul 2;8:104. doi: 10.1186/s40168-020-00878-x (PMC7333401; doi:10.1186/s40168-020-00878-x)
Supplement: Supplementary file 2 — Additional file 1: Table S1 Summary of collection details. The population code (ID), province, city, county, latitude, longitude, and date of the field collections assessed here are provided. Table S2 Specific primers used in PCR for this study. Table S3 Pairwise FST estimates between populations based on a sequence of the mitochondrial COI gene. Population codes are given in Table S1. Table S4 Annual mean temperatures (Bio1) and the annual mean precipitation (Bio12) of the 17 locations obtained from DIVA-GIS 7.5.0. Table S6 Relative abundance of bacterial 16S rRNA genes at the genus level observed for Chinese, Japanese and all populations. Table S7 Effects of factors in the structural equation model (SEM) analysis undertaken on 16 populations where Wolbachia was fixed in the population. Table S10 After Wolbachia was excluded from the Wolbachia-infected adults, the composition of all samples from SAP populations. Table S11 Relative abundance of bacterial 16S rRNA genes at the genus level observed for Wolbachia-infected females (after removal of Wolbachia reads), Wolbachia-uninfected females and all samples. Table S12 Measures of species richness and evenness of SBPH from 10 Wolbachia-infected females (excluding Wolbachia reads) and 9 Wolbachia-free females from the SAP population. [file 40168_2020_878_MOESM1_ESM.docx]

**Supplemental Information for:**

**Recent infection by *Wolbachia* alters microbial communities in wild *Laodelphax striatellus* populations**

Xing-Zhi Duan^1†^, Jing-Tao Sun^1†^, Lin-Ting Wang^1^, Xiao-Han Shu^1^, Matsukura Keiichiro^2^, Yan Guo^1^, Yu-Xi Zhu^1^, Xiao-Li Bing^1^, Ary A. Hoffmann^3^, Xiao-Yue Hong^1*^

^1^Department of Entomology, Nanjing Agricultural University, Nanjing, Jiangsu 210095, China
^2^NARO Kyushu Okinawa Agricultural Research Center, 2421 Suya, Koshi, Kumamoto 861-1192, Japan

^3^School of BioSciences, Bio21 Institute, The University of Melbourne, Victoria 3010, Australia

**Table S1** Summary of collection details. The population code (ID), province, city, county, latitude, longitude, and date of the field collections assessed here are provided.

| ID | Province | City | County | latitude | longitude | Date |
| --- | --- | --- | --- | --- | --- | --- |
| HRB | Heilongjiang | Ha'erbin | Yanshou | 45.48°N | 126.39°E | 08/14/2017 |
| MDJ | Heilongjiang | Mudanjiang |  | 44.55°N | 129.64°E | 07/05/2012 |
| DD | liaoning | Dandong |  | 40.17°N | 124.39°E | 07/16/2012 |
| DZ | Shandong | Dezhou |  | 37.43°N | 116.24°E | 06/22/2012 |
| XX | Henan | Xinxiang |  | 35.32°N | 113.97°E | 06/22/2010 |
| YC | Jiangsu | Yancheng | Xiangshui | 34.11°N | 119.76°E | 06/13/2017 |
| HN | Anhui | Huainan | Fengtai | 32.72°N | 116.67°E | 07/23/2012 |
| XY | Henan | Xinyang |  | 32.16°N | 114.09°E | 06/25/2010 |
| NJ | Jiangsu | Nanjing |  | 32.04°N | 118.87°E | 06/10/2010 |
| LY | Fujian | Longyan |  | 25.12°N | 117.02°E | 05/27/2010 |
| GZ | Guangdong | Guangzhou |  | 23.15°N | 113.35°E | 06/11/2010 |
| SAP | Hokkaido | Sapporo | Hokkaido | 43.03°N | 141.44°E | 08/30/2017 |
| JOE | Hokuriku | Joetsu | Niigata | 37.12°N | 138.27°E | 09/02/2017 |
| KUM | Kanto | Kumagaya | Saitama | 36.17°N | 139.35°E | 09/02/2017 |
| OHD | Chugoku | Ohda | Shimane | 35.15°N | 132.40°E | 07/26/2017 |
| YUK | Kyushu | Yukuhashi | Fukuoka | 33.71°N | 130.95°E | 05/29/2017 |
| ICH | Kyushu | Ichikikushikino | Kagoshima | 31.75°N | 130.25°E | 05/29/2017 |

**Table S2** Specific primers used for PCR in this study.

| Organism | Target | Primer name | Primer sequence (5’→3’) | Product  Size (bp) | Annealing  temp (°C) | Reference |
| --- | --- | --- | --- | --- | --- | --- |
| SBPH | *COI* | COIF  COIR | TCTCATTACATATCGCTGGAGTTAG  GTAGTCTGAATATCGTCGTGGTATT | 887 | 55 | Designed in this study |
| *Wolbachia* | wsp | wsp-F  wsp-R | TGGTCCAATAAGTGATGAAGAAAC  AAAAATTAAACGCTACTCCA | 599 | 52 | Zhao *et al*. [38] |

**Table S3** Pairwise *F*_ST_ estimates between populations based on a sequence of the mitochondrial *COI* gene. Population codes are given in Table S1.

|  | HRB | MDJ | DD | DZ | XX | YC | HN | XY | NJ | LY | GZ | JOE | KUM | OHD | YUK |
| --- | --- | --- | --- | --- | --- | --- | --- | --- | --- | --- | --- | --- | --- | --- | --- |
| MDJ | 0.003 |  |  |  |  |  |  |  |  |  |  |  |  |  |  |
| DD | **0.182** | **0.250** |  |  |  |  |  |  | |  |  |  |  |  |  |
| DZ | **0.130** | **0.194** | -0.022 |  |  |  |  |  | |  |  |  |  |  |  |
| XX | 0.024 | **0.071** | 0.030 | 0.001 |  |  |  |  |  |  |  |  |  |  |  |
| YC | **0.344** | **0.403** | 0.038 | **0.051** | **0.155** |  |  |  |  |  |  |  |  |  |  |
| HN | **0.185** | **0.260** | -0.020 | -0.023 | 0.029 | **0.036** |  |  |  |  |  |  |  |  |  |
| XY | **0.215** | **0.284** | -0.007 | -0.018 | **0.052** | 0.021 | -0.014 |  |  |  |  |  |  |  |  |
| NJ | **0.061** | **0.125** | 0.013 | -0.012 | -0.022 | **0.131** | 0.005 | **0.032** |  |  |  |  |  |  |  |
| LY | **0.151** | **0.218** | 0.002 | -0.022 | 0.015 | **0.058** | -0.010 | -0.024 | 0.005 |  |  |  |  |  |  |
| GZ | **0.390** | **0.457** | 0.034 | **0.059** | **0.175** | -0.019 | 0.038 | 0.026 | **0.151** | **0.072** |  |  |  |  |  |
| JOE | **0.211** | **0.269** | -0.024 | -0.006 | 0.048 | **0.030** | -0.003 | 0.009 | **0.041** | 0.021 | 0.031 |  |  |  |  |
| KUM | **0.511** | **0.568** | **0.130** | **0.165** | **0.287** | 0.020 | **0.140** | **0.129** | **0.271** | **0.186** | 0.009 | **0.106** |  |  |  |
| OHD | **0.269** | **0.332** | 0.012 | 0.005 | **0.089** | 0.024 | 0.026 | -0.004 | **0.064** | 0.021 | **0.043** | 0.020 | **0.141** |  |  |
| YUK | **0.110** | **0.181** | -0.008 | -0.012 | 0.002 | **0.105** | 0.003 | 0.028 | -0.015 | 0.017 | **0.117** | 0.019 | **0.235** | **0.050** |  |
| ICH | **0.422** | **0.486** | **0.066** | **0.083** | **0.208** | -0.022 | 0.070 | 0.044 | **0.182** | **0.096** | -0.023 | **0.061** | 0.011 | **0.041** | **0.150** |
|  |  |  |  |  |  |  |  |  |  |  |  |  |  |  |  |

The bold indicated significant indices (*P* <0.05).

**Table S4** Annual mean temperatures (Bio1) and the annual mean precipitation (Bio12) of the 17 locations obtained from DIVA-GIS 7.5.0.

| ID | Bio1 | Bio12 |
| --- | --- | --- |
| HRB | 3.508 | 565 |
| MDJ | 4.321 | 545 |
| DD | 8.871 | 1040 |
| DZ | 13.863 | 505 |
| XX | 14.963 | 585 |
| YC | 13.946 | 773 |
| HN | 15.754 | 799 |
| XY | 15.429 | 1089 |
| NJ | 15.625 | 1017 |
| LY | 20.633 | 1681 |
| GZ | 22.363 | 1739 |
| SAP | 7.875 | 1140 |
| JOE | 13.458 | 2572 |
| KUM | 14.496 | 1273 |
| OHD | 14.396 | 1784 |
| YUK | 15.842 | 1709 |
| ICH | 16.846 | 2388 |

**Table S6** Relative abundance of bacterial 16S rRNA genes at the genus level observed for Chinese, Japanese and all populations.

| Genus classes | China (%) | Japan (%) | All samples (%) |
| --- | --- | --- | --- |
| *Wolbachia* | 87.9 | 66.4 | 81.2 |
| *Spiroplasma* | 3.55 | 5.00 | 4.01 |
| *Diplorickettsia* | 0.00 | 10.9 | 3.41 |
| *Asaia* | 2.47 | 5.56 | 3.44 |
| *Pantoea* | 1.04 | 2.08 | 1.36 |
| *Herbaspirillum* | 1.03 | 1.05 | 1.03 |
| *unclassified_f__Enterobacteriaceae* | 0.88 | 1.29 | 1.01 |
| *Microbacterium* | 0.81 | 1.23 | 0.94 |
| *Acinetobacter* | 0.10 | 2.33 | 0.80 |
| *unclassified_f__Xanthomonadaceae* | 0.09 | 1.06 | 0.39 |
| others | 2.13 | 3.13 | 2.41 |

**Table S7** Effects of factors in the structural equation model (SEM) analysis undertaken on 16 populations where *Wolbachia* was fixed in the population.

Effects of the latitude on *F*_st_, Bio1 and Bio12:

| Variation | Effect | Effect size ± SE | Z value | *P* |
| --- | --- | --- | --- | --- |
| Genetic differentiation (*F*_st_) | Latitude | 0.387 ± 0.084 | 4.583 | <0.001 |
| Annual mean temperature (Bio1) |  | 0.929 ± 0.033 | 27.800 | <0.001 |
| Annual precipitation (Bio12) |  | 0.001 ± 0.091 | 0.010 | 0.992 |

Effects of the longitude on *F*_st_, Bio1 and Bio12:

| Variation | Effect | Effect size ± SE | Z value | *P* |
| --- | --- | --- | --- | --- |
| Genetic differentiation (*F*_st_) | Longitude | 0.029 ± 0.084 | 0.347 | 0.728 |
| Annual mean temperature (Bio1) |  | -0.053 ± 0.033 | -1.590 | 0.112 |
| Annual precipitation (Bio12) |  | 0.156 ± 0.091 | 1.720 | 0.085 |

Effects of *F*_st_, Bio1, Bio12, Lat and Lon on the microbial Bray Curtis dissimilarity (MCSD):

| Variation | Effect | Effect size ± SE | Z value | *P* |
| --- | --- | --- | --- | --- |
| MCSD | Genetic differentiation (*F*_st_) | 0.257 ± 0.079 | 3.268 | <0.001 |
|  | Latitude | -0.115 ± 0.201 | -0.573 | 0.567 |
|  | Longitude | 0.418 ± 0.074 | 5.643 | <0.001 |
|  | Annual mean temperature (Bio1) | -0.166 ± 0.199 | -0.837 | 0.402 |
|  | Annual precipitation (Bio12) | 0.246 ± 0.073 | 3.358 | <0.001 |

Effects of *F*_st_, Bio1, Bio12, Lat and Lon on the KL divergence (KLD):

| Variation | Effect | Effect size ± SE | Z value | *P* |
| --- | --- | --- | --- | --- |
| KLD | Genetic differentiation (*F*_st_) | 0.230 ± 0.097 | 2.378 | 0.017 |
|  | Latitude | 0.014 ± 0.247 | 0.055 | 0.956 |
|  | Longitude | -0.055 ± 0.091 | -0.597 | 0.550 |
|  | Annual mean temperature (Bio1) | -0.204 ± 0.245 | -0.833 | 0.405 |
|  | Annual precipitation (Bio12) | -0.108 ± 0.090 | -1.195 | 0.232 |

**Table S10** After *Wolbachia* was excluded from the *Wolbachia*-infected adults, the [composition](javascript:;)

of all samples from the SAP population.

| Sample Name | Sequences |
| --- | --- |
| w+1 | 16701 |
| w+2 | 5246 |
| w+3 | 4625 |
| w+4 | 1573 |
| w+5 | 4595 |
| w+6 | 1144 |
| w+7 | 7186 |
| w+8 | 6852 |
| w+9 | 3671 |
| w+10 | 17983 |
| w-1 | 51721 |
| w-2 | 51168 |
| w-3 | 63157 |
| w-4 | 59070 |
| w-5 | 62467 |
| w-6 | 64472 |
| w-7 | 60195 |
| w-8 | 59871 |
| w-9 | 62564 |

**Table S11** Relative abundance of bacterial 16S rRNA genes at the genus level observed for *Wolbachia*-infected females (after removal of *Wolbachia* reads), *Wolbachia*-uninfected females and all samples.

| Genus classes | W+ (%) | W- (%) | All samples (%) |
| --- | --- | --- | --- |
| *Thermus* | 26.5 | 5.58 | 16.6 |
| *Spiroplasma* | 21.6 | 0.84 | 11.8 |
| *Arsenophonus* | 7.76 | 13.4 | 10.5 |
| *Ralstonia* | 17.9 | 0.91 | 9.84 |
| *Prevotella_9* | 0.04 | 15.1 | 7.18 |
| *Prevotellaceae_NK3B31_group* | 0.02 | 5.58 | 2.65 |
| *Lactobacillus* | 1.16 | 3.93 | 2.47 |
| *norank_f__Muribaculaceae* | 2.48 | 1.81 | 2.16 |
| *unclassified_f__Lachnospiraceae* | 0.37 | 4.04 | 2.11 |
| *Megasphaera* | 0.00 | 4.17 | 1.97 |
| *Dialister* | 0.00 | 2.98 | 1.41 |
| *Streptococcus* | 0.09 | 1.98 | 0.98 |
| *Succinivibrio* | 0.00 | 1.68 | 0.80 |
| *Prevotella_2* | 0.00 | 1.30 | 0.61 |
| *Clostridium_sensu_stricto_1* | 0.09 | 1.19 | 0.61 |
| *Selenomonas* | 0.00 | 1.07 | 0.51 |
| *norank_f__Veillonellaceae* | 0.00 | 0.94 | 0.45 |
| *norank_f__AKYH767* | 0.69 | 0.00 | 0.35 |
| others | 21.3 | 33.5 | 27.0 |

**Table S12** Measures of species richness and evenness of SBPH from 10 *Wolbachia*-infected females (excluding *Wolbachia* reads) and 9 *Wolbachia*-free females from the SAP population.

| Samples | Sobs | Shannon | Simpson | Ace | Chao | Coverage |
| --- | --- | --- | --- | --- | --- | --- |
| w+1 | 223 | 3.356 | 0.190 | 723.031 | 402.143 | 0.872 |
| w+2 | 224 | 4.508 | 0.025 | 457.459 | 397.906 | 0.898 |
| w+3 | 34 | 0.667 | 0.760 | 267.682 | 94.000 | 0.976 |
| w+4 | 159 | 3.419 | 0.108 | 230.706 | 202.826 | 0.938 |
| w+5 | 85 | 1.516 | 0.516 | 174.559 | 164.688 | 0.951 |
| w+6 | 121 | 2.611 | 0.176 | 216.559 | 208.652 | 0.938 |
| w+7 | 41 | 1.380 | 0.372 | 92.198 | 101.000 | 0.976 |
| w+8 | 42 | 0.894 | 0.682 | 192.273 | 112.200 | 0.974 |
| w+9 | 56 | 1.496 | 0.414 | 122.637 | 79.400 | 0.974 |
| w+10 | 26 | 0.787 | 0.627 | 175.754 | 77.000 | 0.983 |
| w-1 | 38 | 1.267 | 0.444 | 80.397 | 74.143 | 0.978 |
| w-2 | 54 | 1.347 | 0.414 | 162.009 | 140.667 | 0.961 |
| w-3 | 142 | 3.828 | 0.043 | 204.044 | 203.600 | 0.946 |
| w-4 | 244 | 4.860 | 0.013 | 343.976 | 341.133 | 0.909 |
| w-5 | 161 | 3.805 | 0.052 | 366.133 | 261.100 | 0.925 |
| w-6 | 172 | 3.867 | 0.049 | 365.448 | 284.452 | 0.919 |
| w-7 | 228 | 4.495 | 0.026 | 499.878 | 373.357 | 0.893 |
| w-8 | 154 | 3.725 | 0.053 | 334.910 | 250.464 | 0.929 |
| w-9 | 149 | 3.652 | 0.066 | 249.539 | 270.714 | 0.931 |
